# The prevalence, patterns and predictors of diabetic peripheral neuropathy in a developing country

**DOI:** 10.1186/1758-5996-4-21

**Published:** 2012-05-29

**Authors:** Prasad Katulanda, Priyanga Ranasinghe, Ranil Jayawardena, Godwin R Constantine, M H Rezvi Sheriff, David R Matthews

**Affiliations:** 1 Diabetes Research Unit, Department of Clinical Medicine, Faculty of Medicine, University of Colombo, Colombo, Sri Lanka; 2 Oxford Centre for Diabetes, Endocrinology and Metabolism, University of Oxford, Oxford, UK; 3 Department of Pharmacology, Faculty of Medicine, University of Colombo, Colombo, Sri Lanka; 4 Institute of Health and Biomedical Innovation, Queensland University of Technology, Brisbane, Queensland, Australia

**Keywords:** Diabetes mellitus, Distal peripheral Neuropathy, Prevalence, Sri Lanka, Developing country

## Abstract

Prevalence of diabetes mellitus (DM) has reached epidemic proportions in Sri Lanka. Presently there are studies on the community prevalence of distal peripheral neuropathy (DPN) in Sri Lanka. We describe prevalence, patterns and predictors of DPN in patients with DM in Sri Lanka. Data were collected as part of a national study on DM. In new cases DPN was assessed using the Diabetic-Neuropathy-Symptom (DNS) score, while in those with established diabetes both DNS and Toronto-Clinical-Scoring-System (TCSS) were used. A binary logistic-regression analysis was performed with ‘presence of DPN’ as the dichomatous dependent variable and other independent co-variants. The study included 528 diabetic patients (191-new cases), with a mean age of 55.0 ± 12.4 years and 37.3% were males, while 18% were from urban areas. Prevalence of DPN according to DNS score among all patients, patients with already established diabetes and newly diagnosed patients were 48.1%, 59.1% and 28.8% respectively. Prevalence of DPN in those with established DM as assessed by TCSS was 24% and the majority had mild DPN (16.6%). The remainder of the abstract is based on subjects with established DM. The prevalence of DPN in males and female was 20.0% and 26.4% respectively. The mean age of those with and without DPN was 62.1 ± 10.8 and 55.1 ± 10.8 years respectively (p < 0.001). The majority of those with DPN were from rural-areas (75.3%) and earned a monthly income < Sri Lankan Rupees 12,000 (87.6%). In the binary logistic-regression presence of foot ulcers (OR:10.4; 95%CI 1.8–16.7), female gender (OR:6.7; 95%CI 2.0–9.8) and smoking (OR:5.9; 95%CI 1.4–9.7) were the strongest predictors followed by insulin treatment (OR:4.3; 95%CI 1.3–6.9), diabetic retinopathy (OR:2.7; 95%CI 1.3–5.4), treatment with sulphonylureas (OR:1.8; 95%CI 1.1–3.2), increasing height (OR:1.8; 95%CI 1.2–2.4), rural residence (OR:1.8; 95%CI 1.1–2.5), higher levels of triglycerides (OR:1.6; 95%CI 1.2–2.0) and longer duration of DM (OR:1.2; 95%CI 1.1–1.3). There is a high prevalence of DPN among Sri Lankan adults with diabetes. The study defines the impact of previously known risk factors for development of DPN and identifies several new potential risk factors in an ethnically different large subpopulation with DM.

## Introduction

Diabetes mellitus (DM) has reached epidemic proportions worldwide. Historically, diabetes was considered a disease confined to developed countries and affluent people. However, recent estimates suggest that the prevelence of diabetes is rising globally, particularly in developing countries [1]. Diabetes mellitus has become an important health concern in the South Asian region with an estimated increase in the prevalence of diabetes of over 151% between 2000 and 2030 [1]. Neuropathy is considered the most common micro-vascular complications of both types 1 and 2 diabetes mellitus [2,3]. Neuropathic disorders in diabetes can impair functioning of the central, peripheral and/or autonomic nervous systems [4]. Distal peripheral neuropathy (DPN), also known as diabetic polyneuropathy affects the peripheral nervous system and is by far the most common type of neuropathy seen in DM [5]. The resultant loss of function in peripheral nerves causes loss of protective sensations and impairs patient’s ability to perceive incipient or even apparent ulcerations in the feet. DPN is considered a main risk factor for amputation, and hence a significant cause of morbidity in DM [6]. Although a common and important complication of diabetes, neuropathy has not been studied as often or as extensively as macro-vascular complications or retinopathy and nephropathy [7]. In addition, the reported prevalence estimates vary widely between countries, in part due to the difference in sampling methods and lack of consensus on diagnostic criteria [8]. Hence, for comparative purposes it is important to use studies that utilize similar diagnostic criteria. However, the observed variations in prevalence could partly result from ethnic differences in predisposition and differential exposure to risk factors.

Increasing age, longer duration of diabetes and poor glycaemic control are well recognized risk factors for DPN, while cigarette smoking, retinopathy, hypertension, obesity, hyperlipidaemia and microalbuminuria have also been implicated as potential risk markers [9]. Most prevalence and risk factor studies are from western developed countries, while there is a relative scarcity of data from developing countries, particularly from the South-Asian region [10]. However, an estimated 80% of the global population with diabetes lives in developing countries [1]. The South-Asian population in particular is known to have an increased predisposition for the disease [11]. In addition, several studies have demonstrated that the risk of diabetes related amputations and the prevalence of diabetic foot ulcers in UK is significantly lower in diabetic patients of Asian origin when compared to that of diabetic patients of European origin [12,13]. This reduced risk in Asians was found to be related to the lower incidence of peripheral arterial disease and DPN. Ethnic differences and differential environmental exposure to risk factors in the different populations are other proposed mechanisms. Hence studies aimed at defining the extent of DPN and the associated risk factors in this vast subpopulation of diabetic patients would help to improve preventive strategies.

Sri Lanka is a developing country in the South Asian region with a population of 20.9 million [14]. The prevalence of DM has reached epidemic proportions in Sri Lanka with recent studies demonstrating that one in every five adults aged > 20 years has either diabetes or pre-diabetes [15]. To our knowledge, there are no published data regarding the community prevalence of DPN in Sri Lanka. The present study aims to describe the community prevalence, patterns and predictors of distal peripheral neuropathy among patients with diabetes mellitus in Sri Lanka with a view of identifying differential risk factors, which may lead to improved preventive measures and care for diabetic patients in the South Asia region.

## Materials and methods

### Study population and sampling

Data were collected as part of a wider cross-sectional national study on diabetes conducted in seven of the nine provinces in Sri Lanka between August 2005 and September 2006 (Sri Lanka Diabetes and Cardiovascular Study – SLDCS). In the SLDCS, the research team randomly selected 100 clusters (of 50 adults each) to represent seven out of nine provinces in the country. The sample sizes for each province were determined using a probability-proportional to-size (PPS) technique based on total population of each province. Each cluster was selected by a computer-generated random number list from the ‘Village Office Units’ in each province. Voter registration lists were used to randomly select the first household in each cluster and a uniform criterion was used to select the remaining 49 households. Detailed sampling has been previously reported [15]. Relevant data of 5000 non-institutionalized adults are presented here. The ethical approval for the study was obtained from the Ethical Review Committee, Faculty of Medicine, University of Colombo, Sri Lanka.

### Definitions

Subjects were considered to have ‘diagnosed diabetes’ if they had been previously diagnosed at a government hospital or by a registered medical practitioner. New cases (‘undiagnosed diabetes’) were diagnosed according to the American Diabetes Association [16] and World Health Organization (WHO) criteria [17]. Obesity was defined as a body mass index (BMI) ≥ 27.5 kg/m^2^, based on WHO criteria for Asians [18]. Presence/absence of diabetic retinopathy was determined by standardized dilated ophthalmological examination by two independent ophthalmologists, and classified as background, pre-proliferative, proliferative, advanced retinopathy and maculopathy. Nephropathy was defined as the presence of micro-albuminuria (30 – 299 mg/day) or macro-albuminuria (≥ 300 mg/day), or by review of medical records [19].

### Data collection

In new cases, DPN was assessed using the validated Diabetic Neuropathy Symptom (DNS) Score, while in ‘diagnosed diabetes’ subjects both DNS and the validated Toronto Clinical Scoring System (TCSS) was used [20]. The DNS score derives from the assessment of 4 symptoms, the presence of each symptom is scored 1. DPN is considered to be present if score is between 1 and 4 [21]. The TCSS produces a score derived from the clinical assessment of 6 symptoms, 5 sensory tests, and lower limb reflexes, giving a maximal score of 19. The degree of neuropathy was based on the TCSS score used in a previous study (no neuropathy: ≤5, mild neuropathy: 6–8, moderate neuropathy: 9–11 and severe neuropathy: ≥12) [20]. Sensation was tested on the dorsum and tip of the first toe. Light touch, pain sense, vibration sense and temperature sense were tested using a 10-gram Semmes-Weinsten monofilament, a pinprick, a 128 Hz tuning fork and a cylinder with different temperatures respectively. Tendon reflex was tested by striking the Quadriceps and Achilles tendons with a reflex hammer.

Height was measured using Harpenden stadiometers (Chasmors Ltd, London, UK) to the nearest 0.1 cm, as the maximum distance to the uppermost position on the head from heels, with the individual standing barefoot and in full inspiration. Body weight was measured using a SALTER 920 digital weighing scale (SALTER Ltd, Tonbridge, UK) to the nearest 0.1 kg after an overnight fast and with indoor light clothing. BMI was calculated as weight in kilograms divided by height squared in meters (kg.m-2). Waist circumference was measured midway between the iliac crest and the lower rib margin at the end of normal expiration and hip circumference was measured at the widest level over the greater trochanters using a plastic flexible tape to the nearest 0.1 cm. Seated blood pressure was measured after at least a 10-min rest with Omron IA2 digital blood pressure monitors (Omron Healthcare, Singapore). Fasting venous blood samples were obtained for glucose andlipid estimation from all participants, details of analysis have been previously described [15]. Data on physical activity were collected using the short version of the International Physical Activity Questionnaire. Subjects in the ‘moderate’/‘high’ physical activity categories were considered as being physical active [22].

### Statistical analyses

Data were analysed using SPSS v14 (SPSS Inc., Chicago, IL, USA) statistical software package. The significance of the differences between proportions and means was tested using z-test and Student’s *t*-test or ANOVA respectively. A binary logistic regression analysis was performed in all patients with ‘presence of DPN’ as the dependent variable (0 = DPN absent; 1 = DPN present) and gender (0 = male; 1 = female), sector of residence (0 = Urban, 1 = Rural), household income (0 = > LKR 25,000; 1 = LKR 12,000 – 25,000; 2 = < LKR 12,000), height, triglyceride levels, duration of diabetes, current smoking (0 = no, 1 = yes), current alcohol consumption (0 = no, 1 = yes), retinopathy (0 = absent, 1 = present), nephropathy (0 = absent, 1 = present), Foot ulcers (0 = absent, 1 = present), Obesity (0 = BMI < 27.5, 1 = BMI **≥** 27.5), hypertension (0 = absent, 1 = present), physical activity (0 = active, 1 = inactive), drug treatment (0 = metformin, 1 = sulfonylurea) and treatment with insulin (0 = not treated, 1 = treated) as the independent variables. The variables were selected by a forward selection procedure based on increment of R^2^. In all statistical analyses *p* < 0.05 was considered significant.

## Results

**Figure 1:**
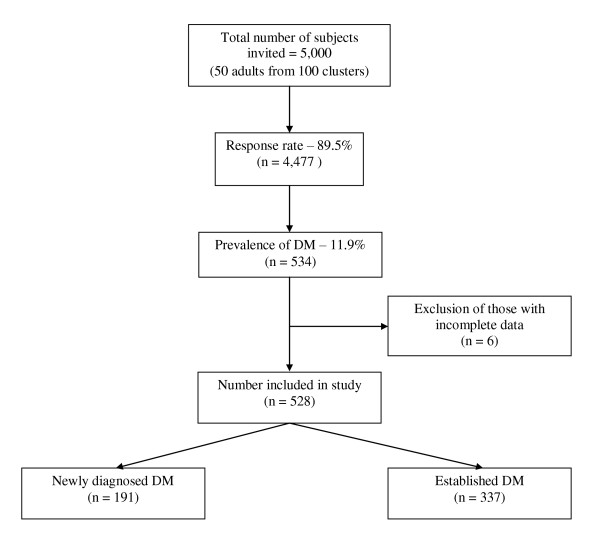
Schematic representation of subject recruitment.

Out of the 5000 invited subjects, 4477 participated in the study (response rate 89.5%). This report is based on 528 subjects (11.9%) with DM after excluding 6 subjects with incomplete data (191 new cases [36.2%]). Details of subject recruitment are presented in Figure 1. The mean age was 55.0 ± 12.4 years, 37.3% were males and only 3 patients were suffering from Type 1 DM. Majority of the subjects were treated with metformin (61.3%) and sulphonylureas (48.4%). Glitazones were used in 4.2%, while insulin was administered in 3.9% of the patients. The prevalence of DPN according to the DNS score among all patients, patients with already established diabetes and newly diagnosed patients was 48.1% (n = 254), 59.1% (n = 199) and 28.8% (n = 55) respectively. The most common symptom among newly diagnosed subjects were burning, aching pain or tenderness of feet (n = 30, 15.7%), followed by numbness of feet (n = 24, 12.6%), prickling sensation of feet (n = 22, 11.5%) and unsteadiness in walking (n = 20, 10.5%).

The remainder of this article is based exclusively on subjects with established DM only (n = 337), in whom both symptoms and signs of neuropathy were evaluated. DPN is considered to be present in all subjects with TCSS > 5. In subjects with established DM the prevalence of DPN according to the TCSS score was 24.0% and a majority was suffering from mild neuropathy (n = 56, 16.6%) (Table 1). In patients with established diabetes the most common symptom was the presence of numbness of feet (n = 125, 37.1%) followed by burning, aching or tenderness of the feet (n = 109, 32.3%), prickling sensation of feet (n = 100, 29.7%) and unsteadiness (n = 86, 25.5%). On clinical examination of subject with established DM, impaired big-toe pin prick sensation was the commonest sensory deficit (n = 134, 39.7%), followed by impaired light touch (n = 129, 38.3%), vibration (n = 88, 26.1%), joint position (n = 56, 16.6%) and temperature sensations (n = 28, 8.3%). Ankle and knee reflexes were absent or diminished in 28.2% (n = 95) and 11.6% (n = 39) of patients respectively. The prevalence of DPN among all, male and female patients was 24.0% (n = 81/337), 20.0% (n = 25/125) and 26.4% (n = 56/212) (males vs. females, p > 0.05). The mean age (±SD) of patients with or without DPN was 62.1 ± 10.8 years and 55.1 ± 10.8 years respectively (p < 0.001). Socio demographic characteristics of subjects with or without DPN are presented in Table 2. A majority of patients with DPN were residing in rural areas (75.3%) and earned a monthly income of < Sri Lankan Rupees (LKR) 12,000 (87.6%). The mean duration of DM among patients with DPN was 7.8 ± 7.1 years, while among patients without DPN it was 5.8 ± 5.0 years (p < 0.01). The presence of foot ulcers were significantly greater in patients with DPN (n = 11, 64.7%) than those without DPN (n = 6, 35.3%) (p < 0.001).

**Table 1:** Socio-demographic characteristics and prevalence of Distal Peripheral Neuropathy among patients with newly diagnosed and established diabetes

	**Diabetes status**		
	Non-diabetic (n = 3943)	Newly diagnosed (n = 191)	Established (n = 337)
Mean age (±SD)	44.9 ± 15.0	51.4 ± 13.5	56.8 ± 11.2
Gender (%) Male	1572 (39.8%)	72 (37.7%)	125 (37.1%)
Female	2377 (60.2%)	119 (62.3%)	212 (62.9%)
DNS score (%)	3123 (79.2%) 488 (12.4%) 175 (4.4%) 102 (2.6%) 55 (1.4%)	136 (71.2%) 29 (15.2%) 14 (7.3%) 9 (4.7%) 3 (1.6%)	138 (40.9%) 73 (21.7%) 54 (16.0%) 49 (14.5%) 23 (6.8%)
TCSS categories No neuropathy (0–5) Mild neuropathy (6–8) Moderate neuropathy (9–11) Severe neuropathy (>11)	NA NA NA NA	NA NA NA NA	256 (76.0%) 56 (16.6%) 24 (7.1%) 1 (0.3%)

* NA – Not applicable.

**Table 2:** Socio-demographic characteristics of diabetic subjects with or without DPN

	**DPN present**	**DPN absent**
Age (±SD)	62.1 ± 10.8	55.1 ± 10.8
Gender (%) Male Female	25 (30.9%) 56 (69.1%)	100 (39.1%) 156 (60.9%)
Sector of Residence (%) Urban Rural	20 (24.7%) 61 (75.3%)	84 (32.8%) 172 (67.2%)
Highest level of Education (%) Primary Secondary Tertiary	32 (39.5%) 49 (60.5%) 0 (0.0%)	52 (20.3%) 192 (75.0%) 12 (4.7%)
Household monthly income (%) < LKR 12,000 LKR 12,000 – 25,000 > LKR 25,000	71 (87.6%) 8 (9.9%) 2 (2.5%)	174 (68.0%) 51 (19.9%) 31 (12.1%)

LKR - Sri Lankan Rupees.

Subjects with DPN were significantly taller and they had a lower body weight, BMI, waist circumference, hip circumference and elevated levels of triglycerides (Table 3 ). However, the strength of association (Spearmans’ correlation) was low (<±0.2) for all of the above factors. No significant difference was observed in the systolic and the diastolic blood pressure, total cholesterol, LDL cholesterol and HDL cholesterol (Table 3). A binary logistic regression was used to identify potential risk factors and associations of DPN in subjects with DM. Presence of foot ulcers, female gender and current smoking were the most significantly and strongly associated factors followed by treatment with insulin, presence of diabetic retinopathy, treatment with sulphonylurea, increasing height, rural sector of residence, higher fasting triglyceride levels, lower household income and longer duration of diabetes after adjusting for age (Table 4). However the presence of hypertension, obesity, nephropathy, alcohol consumption and physical inactivity were not significant predictors of DPN in patients with DM.

**Table 3:** Physical characteristics and biochemical parameters of patients with or without DPN

**Mean (±SD)**				
	DPN present	DPN absent	Correlation coefficient	p value
Height (cm)	155.7 (±9.0)	153.4 (±9.5)	0.167	<0.05
Weight (kg)	54.6 (±11.5)	58.6 (±11.2)	−0.162	<0.01
Body mass index (kg/m^2^)	22.8 (±3.6)	24.2 (±4.0)	−0.150	<0.01
Waist circumference (cm)	83.2 (±11.7)	86.2 (±10.3)	−0.151	<0.05
Hip circumference (cm)	90.9 (±8.9)	93.6 (±8.9)	−0.150	<0.05
Waist-hip ratio	0.92 (±0.08)	0.92 (±0.06)	−0.033	NS
Systolic blood pressure (mmHg)	140 (±25)	138 (±21)	0.030	NS
Diastolic blood pressure (mmHg)	78 (±12)	80 (±12)	−0.082	NS
Total cholesterol (mg/dl)	217.4 (±49.3)	217.1 (±43.2)	−0.007	NS
LDL cholesterol (mg/dl)	138.8 (±38.5)	142.3 (±44.2)	0.013	NS
HDL cholesterol (mg/dl)	47.4 (±9.5)	45.8 (±9.1)	0.063	NS
Triglycerides (mg/dl)	162.1 (±97.4)	138.2 (±67.4)	−0.133	<0.05

**Table 4:** Binary logistic regression of potential risk factors associations with DPN

	**Odds ratio (95% CI)**	**p values**
Gender – male female	reference 6.7 (2.0 – 9.8)	< 0.01
Sector of residence – urban rural	reference 1.8 (1.1 – 2.5)	<0.05
Household monthly income - > LKR 25,000 LKR 12,000 – 25,000 < LKR 12,000	reference 1.5 (1.0 – 2.0) 1.4 (1.0 – 1.8)	<0.05 <0.05
Duration of diabetes	1.2 (1.1 – 1.3)	<0.05
Triglycerides	1.6 (1.2 – 2.0)	<0.05
Height	1.8 (1.2 – 2.4)	<0.05
Current smoking - non-smoker smoker	reference 5.9 (1.4 – 9.7)	<0.05
Alcohol consumption - no yes	reference 3.0 (0.8 – 6.5)	NS
Retinopathy – absent present	reference 2.7 (1.3 – 5.4)	<0.01
Nephropathy – absent present	reference 0.6 (0.1 – 2.5)	NS
Hypertension - absent present	reference 0.8 (0.4 – 1.6)	NS
Obesity - BMI <27.5 BMI > =27.5	Reference 1.0 (0.5 – 1.9)	NS
Foot ulcers – absent present	Reference 10.4 (1.8 – 16.7)	<0.01
Physical activity - inactive active	Reference 0.9 (0.4 – 2.3)	NS
Drug treatment - metformin sulphonylurea	Reference 1.8 (1.1 – 2.5)	<0.05
Insulin – not treated with insulin treated with insulin	Reference 4.3 (1.3 – 6.9)	<0.05

LKR - Sri Lankan Rupees.

## Discussion

DPN is a common complication of DM. This is the first comprehensive report on DPN among DM patients from a nationally representative community based sample from Sri Lanka. The Western, Southern, Sabaragamuwa, Uva, North-Western, Central and North-Central provinces were included while the Northern and Eastern provinces affected by the war had to be excluded. The prevalence of DPN according to the DNS and the TCSS scores was 48.1% and 24.0% respectively. The diagnosis of DPN is complicated and requires the assessment of multiple features of neuropathy as DPN affects a variety of nerve fibres. [23]. For full classification the San Antonio Conference on diabetic neuropathy recommends the assessment of at least one measure from each of the following categories: neuropathic symptoms, clinical examination, electro-diagnostic tests, quantitative sensory tests and autonomic function tests [24]. Thus, the TCSS which incorporates both assessment of symptoms and signs can be considered more accurate for comparative studies. Sri Lankan community-based prevalence of DPN is comparable with that reported from India, Bangladesh and UK using similar diagnostic criteria. However, the prevalence of DPN in Sri Lanka is significantly lower than in other regional, developing and developed countries (Table 5), differences in diagnostic methodologies could partially account for this difference. Ethnic differences in predisposition and differential exposure to risk factors are other probable mechanisms. Most studies are based on either in-hospital patients or follow-up patients attending diabetic clinics. Recent reviews have noted that “knowledge on the epidemiology of DPN is compromised by the lack of large population-based studies” [8].

**Table 5:** Prevalence of DPN in regional and other developed countries

**Country**	**Prevalence of DPN (%)**		
	**All**	**Male**	**Female**
Sri Lanka	24.0%	20.0%	26.4%
Bangladesh [10]	19.7%	20.9%	18.7%
India [25]	29.0%	27.9%	30.8%
Pakistan [26]	39.6%		
UK [27]	28.5%	28.5%	28.5%
China [28]	32.0%		
Iran [29]	51.7%	49.9%	51.7%
North-Africa [30]	41.0%	38.5%	43.9%

In the present study there was a significant association between the use of insulin and presence of DPN. Some have postulated that exogenous insulin therapy in T2DM might be associated with DPN through an exacerbation of obesity, fluid retention, hypertension and hyperlipidemia [31]. However, randomized clinical trials have shown that intensive insulin therapy can prevent or delay the development of DPN compared to conventional insulin therapy [32]. Another possible explanation for the association of insulin use with the prevalence of DPN in this cohort could be that insulin use indicates beta cell failure in this group of patients and may reflect a later stage in the natural history of diabetes or a greater severity. Another interesting observation is the trend towards DPN protection observed in the patients treated with metformin as opposed to sulphonylureas. It is known that metformin has multiple effects with direct vascular implications, such as improvement in lipid profile, prevention of oxidative stress-induced endothelial cell death and direct neuro-protective effects via inhibition of oxidative stress-related apoptotic cell death in primary neurons [33]. On the other recent animal studies have shown that blockage of K^+^ channels in neurons by sulphonylureas may selectively potentiate neurotoxicity [34].

The association of elevated fasting triglycerides with DPN supports the emerging idea that hyper-triglyceridaemia contributes to the development and the progression of diabetic neuropathy [35]. Elevated serum triglycerides are commonly associated with insulin resistance and represent a valuable clinical marker of the metabolic syndrome and the resultant atherogenic potential could contribute towards the progression of DPN [36]. In addition, Schwann cell lipid metabolism has also been found to be abnormal in DPN, and hence elevated triglycerides may represent a blood marker of the pathological changes in the myelin structure of nerve [37]. Thus, correction of elevated triglycerides with dietary control or drug treatment may have an ameliorative effect on the development and the progression of DPN. The significant association observed between DPN and smoking may at least in part be secondary to the vascular effects smoking, as there has been increasing evidence of the importance of microvascular factors in the pathogenesis of DPN [38].

The presence of DPN was associated with rural sector of residence and lower household income. A possible explanation for the phenomenon could be that poor people are less likely to use health services, which might result in delayed diagnosis and poor control of DM [39]. Previous studies have reported that metabolic control of DM was worse in patients with a lower socio-economic status [40]. In addition, the increased risk in rural sector residents could be due to lack of access to the better health care facilities available to residents in urban areas. The association of increased height with DPN indicates that increased stature has a generalized adverse effect on peripheral nerve function. The increased nerve length in taller people is associated with greater axon surface area. Therefore, persons with longer nerves (and thus a larger total axon surface area) may be at greater risk for neurologic impairment when exposed to otherwise equivalent hazards. Greater leg length might also be associated with a prolonged time requirement for the complete regeneration of any injured nerve [41]. Finally, in the present study female gender was associated with an increased risk of developing DPN. The finding is not in agreement, with cross-sectional data from other studies such as the Diabetes Control and Complications Trial, where neuropathy at baseline were significantly more likely in males [42]. Further studies are required to determine if there are true gender related differences in the risk of developing DPN.

Several variables such as obesity, presence of hypertension, high serum cholesterol and alcohol consumption identified as predictors of DPN in other populations did not emerge as independent predictors in the present study. According to our results subjects with DPN had a significantly lower body weight, BMI and waist circumference. In addition presence of obesity was not a significant risk factor for DPN in logistic regression analysis. Several studies from Asian countries have also reported similar results or no association between obesity and presence of DPN [10]. Hence, further studies are required to define the role of body weight in DPN in the Asian population. We also found little evidence for an important role of blood pressure, even though in other studies hypertension emerged as a strong risk factor [43]. The reasons for these discrepant findings are not clear. Our study has several limitations. The “duration of diabetes” as measured in this study might not reflect the true duration of the disease but the time since diagnosis and actual diabetes onset might precede diagnosis by several years. We were also unable to assess the impact of glycaemic control on presence of DPN, as data on HbA1c values were not available for the majority of the population.

## Conclusions

The present study defines the impact of previously known risk factors for the development of diabetic peripheral neuropathy in the South-Asian population, while in addition identifies several new potential risk factors of importance in this ethnically different large sub population with diabetes. The examination of these patients in the future may lead to identification of factors which have led to the development of neuropathy or the progress of established neuropathy, which will enable risk reduction strategies to be developed.

## Competing interests

The author(s) declare that they have no competing interests.

## Authors’ contributions

PK, GRC, DRM and MHRS made substantial contribution to conception and study design. PK and GRC were involved in data collection. RJ and PR were involved in refining the study design, statistical analysis and drafting the manuscript. RJ, PR, MHRS, GRC and PK critically revised the manuscript. All authors read and approved the final manuscript.
